# Whole-Genome Sequencing of *Salmonella* Mississippi and Typhimurium Definitive Type 160, Australia and New Zealand

**DOI:** 10.3201/eid2509.181811

**Published:** 2019-09

**Authors:** Laura Ford, Danielle Ingle, Kathryn Glass, Mark Veitch, Deborah A. Williamson, Michelle Harlock, Joy Gregory, Russell Stafford, Nigel French, Samuel Bloomfield, Zoe Grange, Mary Lou Conway, Martyn D. Kirk

**Affiliations:** The Australian National University, Acton, Australian Capital Territory, Australia (L. Ford, D. Ingle, K. Glass, M.D. Kirk);; The University of Melbourne at the Peter Doherty Institute for Infection and Immunity, Melbourne, Victoria, Australia (D. Ingle, D.A. Williamson);; Department of Health, Hobart, Tasmania, Australia (M. Veitch, M. Harlock);; Victorian Department of Health and Human Services, Melbourne (J. Gregory);; Queensland Department of Health, Brisbane, Queensland, Australia (R. Stafford);; New Zealand Food Safety Science and Research Centre, Manawatu-Wanganui, New Zealand (N. French);; Massey University, Manawatu-Wanganui (N. French); Quadram Institute, Norwich, UK (S. Bloomfield);; University of California Davis–Davis, California, USA (Z. Grange);; Department of Primary Industries, Parks, Water and Environment, Hobart (M.L. Conway)

**Keywords:** *Salmonella*, genomics, epidemiology, humans, animals, zoonoses, birds, drinking water, Australia, Tasmania, New Zealand, bacteria, whole-genome sequencing, food safety

## Abstract

We used phylogenomic and risk factor data on isolates of *Salmonella enterica* serovars Mississippi and Typhimurium definitive type 160 (DT160) collected from human, animal, and environmental sources to elucidate their epidemiology and disease reservoirs in Australia and New Zealand. Sequence data suggested wild birds as a likely reservoir for DT160; animal and environmental sources varied more for *Salmonella* Mississippi than for *Salmonella* Typhimurium. Australia and New Zealand isolates sat in distinct clades for both serovars; the median single-nucleotide polymorphism distance for DT160 was 29 (range 8–66) and for *Salmonella* Mississippi, 619 (range 565–737). Phylogenomic data identified plausible sources of human infection from wildlife and environmental reservoirs and provided evidence supporting New Zealand–acquired DT160 in a group of travelers returning to Australia. Wider use of real-time whole-genome sequencing in new locations and for other serovars may identify sources and routes of transmission, thereby aiding prevention and control.

Nontyphoidal *Salmonella enterica* subsp. *enterica* causes substantial illness and death throughout the world (*1*,*2*). In Australia, rates of notified infection are higher than in other high-income countries (*3*). Preventing infection by understanding sources and routes of transmission and controlling outbreaks rapidly is key to reducing the rate of salmonellosis in Australia. Whole-genome sequencing (WGS) is increasingly being used as a tool to help with prevention and control by investigating the relationship between isolates, sources of infection, and routes of transmission (*4*). Evidence shows that WGS is useful in foodborne nontyphoidal *S. enterica *outbreak detection and control (*5**–**7*).

On mainland Australia (Figure 1), *Salmonella* Typhimurium is the most commonly notified nontyphoidal *S. enterica* serovar. In contrast, *Salmonella* Mississippi is the most commonly notified nontyphoidal *S. enterica* serovar infecting residents of the island state of Tasmania, where 2.1% of the population of Australia resides (*3**,**8*). Most persons with *Salmonella* Mississippi who are residents of mainland Australia have traveled to Tasmania or one of several Pacific Islands to which *Salmonella* Mississippi was endemic during their exposure period (*9*,*10*). In Tasmania, *Salmonella* Mississippi has been isolated from wildlife, and some evidence indicates that human infections might result from environmental transmission more frequently than from foodborne transmission (*9*,*11*). A case–control study in Tasmania during 2001–2002 found that case-patients were more likely than controls to have had indirect contact with native birds, consumed untreated drinking water, and traveled within the state (*9*), although the sources of infection and vehicles of transmission are still largely unknown.

**Figure 1:**
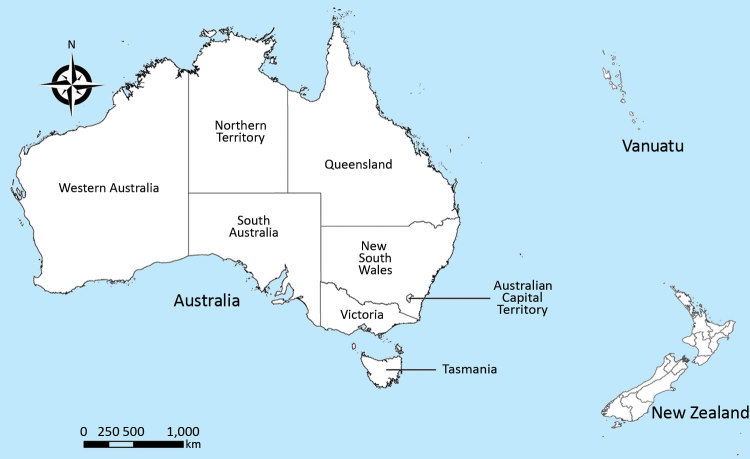
Relative locations of Australia, New Zealand, and Vanuatu.

*Salmonella* Typhimurium definitive type 160 (DT160) has more recently emerged in Tasmania, while remaining rare in the rest of the country. In 2008, ten years after its emergence in humans in New Zealand, the first locally acquired case of DT160 was reported in Tasmania; unusual sparrow (*Passer domesticus*) deaths were observed in the same area in 2009, consistent with sparrow deaths in New Zealand in 2000 (*12**–**15*). Since then, DT160 has affected ≈50 Tasmania residents and ≈3,000 New Zealand residents and has been associated with wild bird deaths in both countries (*16**–**18*). Although a case–control study conducted in 2001 in New Zealand suggested that handling of dead wild birds, contact with persons with diarrheal illness, and ingestion of fast food were associated with illness (*12*), the relationship between the Tasmania and New Zealand DT160 infections and the relationship between animal and human isolates in Tasmania is unknown. Accordingly, we aimed to use these 2 nontyphoidal *S. enterica* serovars as case studies to investigate how epidemiologic and genomic data can be integrated to better understand the geographic niche and transmission pathways of these organisms to subsequently improve prevention and control strategies.

## Methods

### Ethics Considerations and Data Sources

The Australian National University Human Research Ethics Committee (2016/269) granted ethics approval for this project. We used data from the Australian National Notifiable Diseases Surveillance System (*18*) and the New Zealand Enteric Reference Laboratory (*19*) to examine trends in *Salmonella* Mississippi and DT160 in Australia and New Zealand. Population denominator data were obtained from the Australian Bureau of Statistics (*8*) and Statistics New Zealand (*20*). We used postcode of residence to determine Australian state or territory. Travel information was not available, so postcode might not represent place of acquisition for all notifications.

We collated epidemiologic data obtained through enhanced surveillance of cases of *Salmonella* Mississippi and DT160 that were notified to the Department of Health and Human Services in Tasmania, the Department of Health and Human Services in Victoria, and Queensland Health. Case-patients were interviewed at the time of notification using a standardized questionnaire, and this information was collected under each jurisdiction’s public health legislation. For human isolates for which sequence data were available, we obtained the following case data fields from these questionnaires: type of case (sporadic, household, cluster, outbreak); hospitalization (yes/no); symptoms; travel; close contact with farm animals, native animals, birds, or pets (yes/no and type); lives on a rural property (yes/no); bushwalking or camping (yes/no); water source (public, private, or bottled); gardening (yes/no); swimming (yes/no); other risk factors (free text). Data on risk factors were collected for the week before illness onset.

### Isolate Selection

For *Salmonella* Mississippi, we selected 34 human isolates from Tasmania residents and 28 human isolates from residents of other states and territories in Australia that were referred for characterization to the Microbiological Diagnostic Unit Public Health Laboratory (MDU PHL) in Melbourne during January 1, 2011–December 31, 2015, for WGS (Appendix). We also selected all viable isolates with a recorded source from 42 animal sources and 18 environmental sources in the MDU PHL collection with an isolation date from January 1, 2000, through December 31, 2016; these isolates were all from Tasmania. For DT160, all viable isolates held in the MDU PHL collection at the beginning of 2016 were included in genomic analysis.

### Sequencing and Bioinformatics

MDU PHL performed DNA extraction and WGS for the Australia isolates. Sequence libraries were prepared using NexteraXT and sequenced on the Illumina NextSeq500 platform (Illumina, https://www.illumina.com) with 150 bp paired-end reads. Reads are available from the National Center for Biotechnology Information Sequence Read Archive (PRJNA319593). *Salmonella* Typhimurium L2 (accession no. NC003197 [https://www.ncbi.nlm.nih.gov/nuccore/NC_003197.2]) was used as a reference for DT160, and because complete genomes were not publicly available, we used a local reference (AUSMDU00020775) for *Salmonella* Mississippi by assembling 1 of the isolates in this analysis (Appendix). We included 10 publicly available draft assemblies from 2011 through 2013 from New Zealand and the United States (Appendix) in the *Salmonella* Mississippi analysis (*21*,*22*) and 106 publicly available DT160 genomes from 1992 through 2012 from humans, wild birds, poultry, and bovine sources in New Zealand in the DT160 analysis (*16*).

*Salmonella* Mississippi and DT160 genomes were analyzed separately using Nullarbor version 2 (https://github.com/tseemann/nullarbor). Short-read data of the isolates were mapped to the reference using Snippy version 4.0-dev2 (https://github.com/tseemann/snippy). The 10 publicly available *Salmonella* Mississippi draft assemblies were also mapped to the *Salmonella* Mississippi reference using Snippy , with the –ctgs parameter that enables mapping of assembly contigs to a reference. A core genome alignment was produced using Snippy-core (v4.0-dev 2), and the resulting full alignment was then filtered for recombination using Gubbins (*23*) using the weighted Robinson-Foulds method to estimate convergence with an initial 10 iterations. The resulting recombination-filtered core genome alignment of 8,573 bases for *Salmonella* Mississippi and 2,203 bases for DT160 was then passed to RAxML version 8.2.11 (*24*) to infer maximum-likelihood (ML) phylogenetic trees, using the generalized time-reversible model with a γ-distribution to model site-specific rate variation and ascertain bias correction. For each analysis, we used 3 independent runs with 1,000 bootstrap pseudoreplicates to assess branch support, with the phylogenetic trees with the highest support across the 3 runs used as the final tree for each analysis. The pairwise single-nucleotide polymorphism (SNP) distances between isolates were calculated from the recombination-filtered core genome alignment using *afa-pairwise.pl* within Nullarbor. De novo genome assemblies were generated using SPAdes version 3.12.0 (*25*), and the presence of known antimicrobial resistance genes was investigated using ABRicate (https://github.com/tseemann/abricate) in conjunction with the genome assemblies and the National Center for Biotechnology Information antimicrobial resistance database with a minimum coverage of 90% and minimum identity of 90%.

### Data Analysis

We collated epidemiologic and SNP data in Microsoft Excel 2013 (https://www.microsoft.com) and performed descriptive analyses in Stata SE 14 (https://www.stata.com). We used sequence data to explore hypotheses about the epidemiologic relatedness of isolates. We compared risk factors between DT160 and *Salmonella* Mississippi cases using a 2-sample test of proportions. Based on SNPs between isolates of each serovar with known epidemiologic links (household or epidemiologic cluster), we considered isolates within 8 SNPs of each other for DT160 and 10 SNPs of each other for *Salmonella* Mississippi a putative phylogenetic cluster for further investigation.

## Results

### *Salmonella* Mississippi

**Figure 2:**
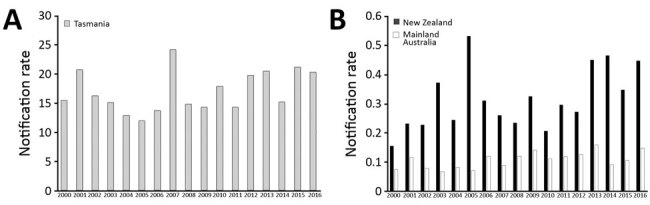
Notification rates for *Salmonella enterica* serovar Mississippi, Tasmania (A) and mainland Australia and New Zealand (B), 2000–2016. Rates are per 100,000 population.

During January 1, 2000–December 31, 2016, the median annual notification rate of *Salmonella* Mississippi in Tasmania was 15 cases (range 12–24 cases) per 100,000 population, compared with a notification rate of 0.11 cases (range 0.007–0.16 cases) per 100,000 population on mainland Australia and 0.3 cases (range 0.16–0.47 cases) per 100,000 population in New Zealand (Figure 2). In Australia, 934 (50.5%) of 1,851 notifications occurred in female patients. Children 0–4 years of age were the most frequently notified age group (458 [24.7%]). Notification rates were higher in warmer months, similar to those for other non-Typhimurium serovars (*26*).

**Figure 3:**
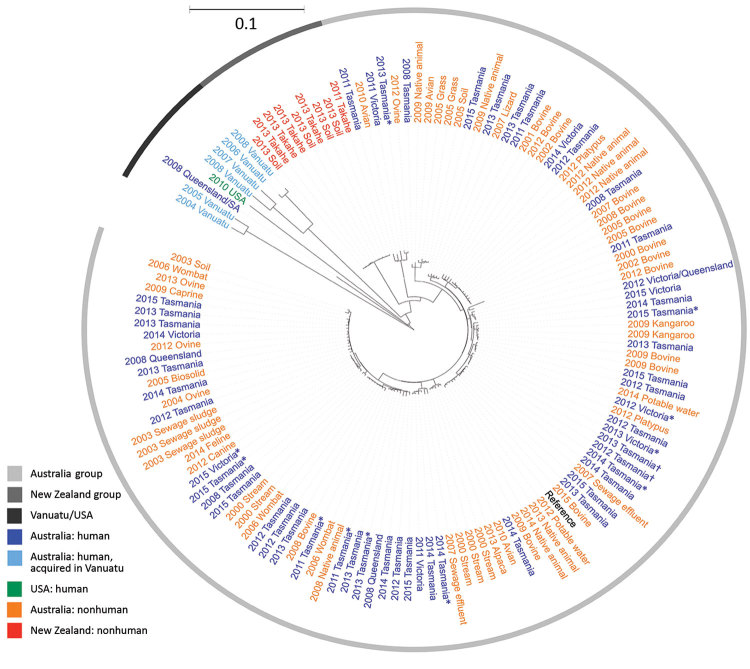
Maximum-likelihood phylogeny of 132 sequenced *Salmonella enterica* serovar Mississippi isolates from Australia and New Zealand and reference isolates, inferred from 8,573 core single-nucleotide polymorphisms. Nodes are labeled with isolation year, isolate source if nonhuman (all from Tasmania), and Australia state of acquisition or residence if human. Tree visualized with iTOL (https://itol.embl.de) and midpoint rooted. Scale bar indicates nucleotide substitutions per site. *State of residence was used instead of state of acquisition because no travel data were available; †investigated as part of an epidemiologic cluster.

For sequenced *Salmonella* Mississippi isolates, the ML tree showed that the isolates from Vanuatu, United States, and New Zealand were distinctly different from the Australia isolates (Figure 3); median SNP distance was 619 (range 565–737) between Australia and New Zealand isolates (Appendix Figure 1) and 1,625 (range 963–1,923) between Australia and Vanuatu or US isolates. An isolate from a 17-week-old mainland Australia resident with no history of overseas travel grouped on the phylogenetic tree with the isolates acquired in Vanuatu. Within the large Australia group, the 114 isolates were diverse; median SNP distance was 169 (range 3–649). Of these Australia isolates, 24 (21.1%) of 114 were within 10 SNPs of another isolate and grouped into 8 phylogenetic clusters. The Australia human isolates grouped with Australia animal and environmental isolates over several years. We observed considerable genetic diversity between isolates from various animal sources. For example, isolates from wombats were a median of 87 (range 44–97) SNPs apart, and isolates from bovines were a median of 140 (range 10–201) SNPs apart. We did not detect any antimicrobial resistance genes in these isolates, except for 1 human isolate from Tasmania that had the *bla*_TEM-1_ gene, which mediates resistance to ampicillin.

Travel data were available for 51 (82%) of 62 of case-patients in Australia residents for which an isolate was sequenced. Of these, 6 (12%) reported international travel to Vanuatu and 19 (37%) reported domestic travel during their incubation period; 8 traveled from mainland states to Tasmania, 9 traveled within Tasmania, 1 traveled from Queensland to South Australia, and 1 traveled from Victoria to Queensland. Three Tasmania isolates investigated as an epidemiologic cluster clustered genetically and temporally with an isolate from Victoria, for which no epidemiologic data were available. Although 2 other phylogenetic clusters included >1 human isolate, epidemiologic data were limited for these cases, and the infections were not clustered in time. Although not statistically significant, among case-patients who resided in or were known to have acquired infection in Tasmania and answered risk factor questions about exposures, a higher proportion of *Salmonella* Mississippi than DT160 case-patients reported drinking water from an untreated raw water source (i.e., tank, spring, or bore) (61% vs. 40%; p = 0.1) and camping (8% vs. 0%; p = 0.07) (Appendix Table 4). A similar proportion of DT160 and *Salmonella* Mississippi cases reported bushwalking (8% vs. 7.5%; p = 0.94), gardening (16% vs. 22%; p = 0.56), and swimming (16% vs. 18%; p = 0.84) exposures. Of the 13 case-patients who resided in or acquired their infection in an Australia state other than Tasmania, 4 (31%) reported eating oysters during the exposure period, including 2 cases in a phylogenetic cluster; however, not all case-patients were asked specifically about oysters.

### *Salmonella* Typhimurium DT160

**Figure 4:**
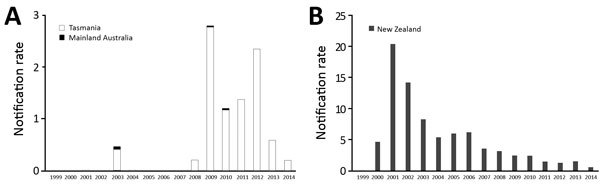
*Salmonella enterica* serovar Typhimurium definitive type 160 notification rate, Tansmania and mainland Australia (A) and New Zealand (B), 1999–2014. Rates are per 100,000 population.

A total of 61 DT160 cases in Australia residents were notified during 1999–2014. The 12 infections reported in Australia residents before 2008 were believed to be acquired overseas, including cases on an Australia–New Zealand cruise in 2003. Most Australia DT160 notifications had a postcode of residence in Tasmania, where the median annual rate per 100,000 population of DT160 in the 9 years from 1999 to 2007 was 0 cases and in the 7 years from 2008 to 2014 was 1.2, peaking at 2.8 in 2009, when 14 cases occurred. In New Zealand, the median annual rate per 100,000 population of DT160 in the 9 years from 1999 to 2007 was 6, peaking at 20.4 in 2001, when 791 cases occurred. In the 7 years from 2008 to 2014, the median annual rate was 1.6 per 100,000 population (Figure 4). Of all 61 Australia notifications during 1999–2014, a total of 34 (56%) occurred in females, and children aged 0–4 years were the most frequently notified age group (14 [23%]). Because of the small number of cases, we found no clear seasonal pattern.

**Figure 5:**
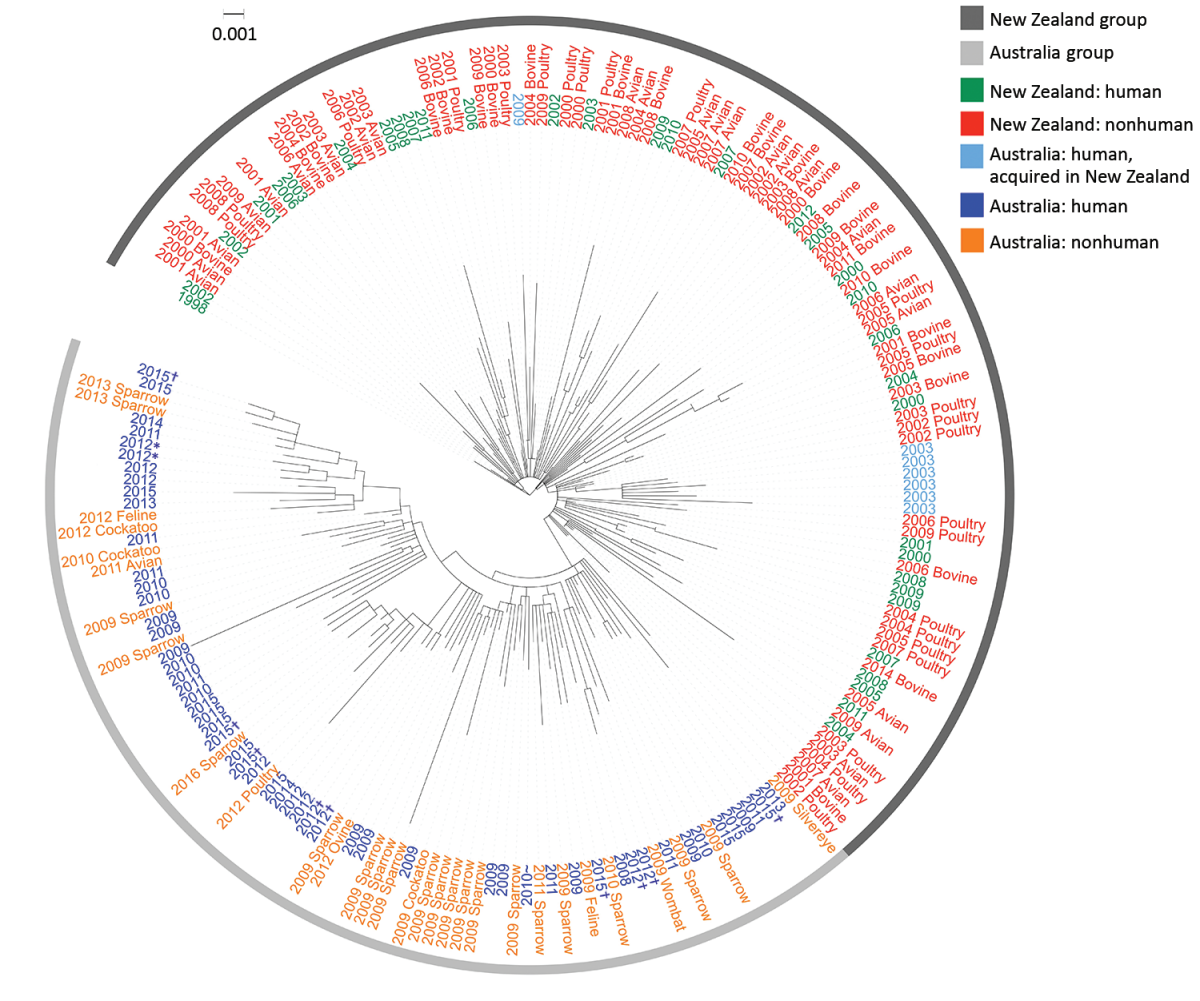
Maximum-likelihood phylogeny of 198 sequenced *Salmonella enterica* serovar Typhimurium definitive type 160 isolates from Australia and New Zealand and reference isolates, inferred from 2,203 core single-nucleotide polymorphisms, Australia and New Zealand. Nodes are labeled with isolate type and isolation year. All Australian isolates are from Tasmania unless specified otherwise. Figure created with iTOL (https://itol.embl.de). Scale bar indicates nucleotide substitutions per site. *Specimens from the same person. †Investigated as part of an epidemiologic cluster. ‡Acquired in New South Wales.

Of Australia isolates, we sequenced 62 human and 30 animal isolates (20 from sparrows). The ML tree of Australia and New Zealand isolates showed 2 distinct groups; 1 comprised isolates from humans and animals in Australia, and 1 comprised humans and animals from New Zealand and the 7 Australia residents who had reported travel to New Zealand (Figure 5). The median pairwise SNP difference between the Australia and New Zealand groups was 29 (range 8–66), and the median pairwise SNP difference within each group was 21 (Australia, range 2–56; New Zealand, range 0–55) (Appendix Figure 4). Within the Australia group, 45 (53%) of 85 isolates were within 8 SNPs of another isolate, and the isolates grouped into 8 phylogenetic clusters. Of these 8 phylogenetic clusters, 3 contained >2 isolates; isolates from humans and birds over several years clustered. No known antimicrobial resistance genes were detected among any of the isolates.

Epidemiologic risk factor data were available for 55 (90%) of the 61 DT160 cases in Australia residents from the 62 sequenced human isolates (1 person contributed 2 isolates, 13 days and 7 SNPs apart). Of these 55 persons, 7 (12%) of 59 acquired their infection in New Zealand, 6 in 2003 on an Australia–New Zealand cruise, and 1 in 2009. All others were residents of, or had traveled to, Tasmania, except for 1 case-patient, who was a resident of New South Wales and had no reported travel outside the state. Two separate household clusters were investigated in 2012 and were phylogenetically clustered. In 2015, five isolates were investigated as part of an epidemiologic cluster; however, no epidemiologic link was found, and they were subsequently found not to be phylogenetically clustered (median SNPs 25.5, range 9–33). A higher proportion of DT160 case-patients than *Salmonella* Mississippi case-patients reported direct contact with wild or domestic animals (88% vs. 68%; p = 0.04) (Appendix Table 4).

## Discussion

Phylogenomics plays a valuable role in identifying plausible sources of *Salmonella* infection from wildlife and environmental reservoirs. For both serovars considered in this article, the integration of clinical and genomic data enhanced existing evidence on source reservoirs by showing that human and animal or environmental isolates were genetically interspersed. Phylogenomic analysis revealed genetic diversity and persistence of *Salmonella* Mississippi strains in the environment and animals, suggesting it is endemic with a broad range of host reservoirs in Tasmania that is persisting over time. As in other countries (*16*,*27**–**29*), genomic analysis provided evidence that wild birds are a source of human infection with DT160. No characterized antimicrobial resistance genes were detected in any of the DT160 draft genome sequences, suggesting low or no antimicrobial resistance, consistent with other *Salmonella* strains found in wild birds (*30*,*31*).

In contrast to *Salmonella* Mississippi, *Salmonella* Typhimurium DT160 isolates from Australia and New Zealand were similar, suggesting possible recent trans-Tasmania transmission of DT160 through wildlife, as well as its potential to spread from Tasmania to the Australia mainland. Further, the inferred population structure of these Australia strains provided evidence for this hypothesis of import and subsequent microevolution within the Australia strains. In New Zealand, DT160 has been transmitted between multiple hosts, and humans have been infected from multiple sources (*16*). Other *Salmonella* serovars with wild bird reservoirs have been transmitted to cattle, pigs, sheep, and poultry (*32*). The incidence of human infection in Australia most likely would increase if DT160 were to become established in local animal food sources.

Epidemiologic evidence that 88% of DT160 case-patients had direct animal contact and the close genetic relatedness between Australia human and animal isolates suggest that DT160 in Tasmania is predominantly a locally acquired zoonotic infection. Control measures should therefore focus on promoting hand hygiene after contact with wild birds and other animals, keeping food preparation and eating areas free from birds, treating drinking water that is accessible to birds and other animals, and appropriately cleaning and maintaining birdfeeders (*32*,*33*). In addition to monitoring the effect of such measures, continued isolation, identification, and WGS of DT160 isolates from humans, animals, and the environment could be used to monitor emergence in other settings that pose particular risks to the food supply and provide early warning of the need for specific control measures.

Risk factors for *Salmonella* Mississippi are less evident, although the proportion of case-patients who reported drinking water from a private source (61%) was similar to that of an Australia case–control study of *Salmonella* Mississippi (63% of case-patients vs. 23% of controls reported drinking any untreated water; adjusted odds ratio 6.13, 95% CI 3.19–11.76) (*9*). The range of animal and environmental sources and the high genetic diversity make identifying control strategies difficult. Because genomic analyses of WGS data have detected clusters when epidemiologic links are obscured (*7*), prospective WGS of case isolates and integration of WGS data from human, animal, and environmental isolates could help to identify putative clusters for targeted epidemiologic investigation. Source attribution studies might enable quantification of the contribution of raw water as a vehicle for *Salmonella* Mississippi infection in Tasmania.

One limitation of this study is that our sampling frame for sequencing and analysis might not have produced a representative sample of all infections; however, we tried to maximize variability of sources and isolation dates. Although epidemiologic risk factor data were incomplete for sequenced human cases, sufficient data were available for us to generate hypotheses that could be further investigated. Our case–case method is not as robust as a case–control study with a neutral control group, but we believe it is a reasonable point of comparison, emphasizing the difference in risk between these 2 serovars. Some more recent Australia DT160 notifications might not have been captured by the national notification system because phage typing for *Salmonella* Typhimurium is being phased out across Australia. However, we believe this omission would be a small number because phage typing continued in most of the country until 2016 (M. Valcanis, MDU PHL, pers. comm., 2018 Oct 10). Without phage typing, and as the use of WGS for *Salmonella* surveillance becomes more routine, it will be difficult to compare new isolates with historically phage-typed isolates that have not been sequenced.

We used SNP thresholds based on known epidemiologic clusters to define putative phylogenetic clusters and to examine epidemiologic risk factors. Because SNPs depend on the reference genome and the isolates in the analysis, SNP thresholds for cluster analysis are likely to differ according to context. Local references were unavailable for both serovars in this study. We assembled a reference for *Salmonella* Mississippi using an isolate in this study, and the *Salmonella* Typhimurium isolate used was a median of 899 (range 883–921) SNPs from the DT160 isolates in our study. A closer reference for DT160 might have provided higher resolution of the relatedness of isolates. Few international *Salmonella* Mississippi genomes were publicly available. Therefore, the relationship we found between Australia and New Zealand isolates might not be representative of all *Salmonella* Mississippi isolates in these 2 countries. Although beyond the scope of this study, identifying the most recent common ancestor using Bayesian phylogeographic analyses would improve our understanding of endemic strains such as *Salmonella* Mississippi and the translocation of emerging strains such as DT160.

Wildlife can contribute to substantial rates of endemic and epidemic infection from *Salmonella*. For these 2 *Salmonella* serovars with wild animal and environmental reservoirs in Australia and New Zealand, WGS combined with epidemiologic risk factor data provided some evidence for prevention and control efforts demonstrating the potential benefits of using WGS for prospective *Salmonella* surveillance. Real-time sequencing of these strains could help monitor emergence and identify clusters, enabling epidemiologists to more accurately identify common risk factors and aid in source attribution. Local references, publicly available international genomes, phylogeographic analyses, additional tools to define a WGS cluster, and source-assigned case–control studies would improve our understanding of the epidemiology of these 2 *Salmonella* serovars in this region.

Appendix Additional and methods for study of whole-genome sequencing of *Salmonella enterica* subtypes Mississippi and Typhimurium DT160, Tasmania, Australia, and New Zealand.
