# Supplementary material for: Whole-Genome Sequencing of Salmonella Mississippi and Typhimurium Definitive Type 160, Australia and New Zealand
Source: Emerg Infect Dis. 2019 Sep;25(9):1690–7. doi: 10.3201/eid2509.181811 (PMC6711244; doi:10.3201/eid2509.181811)
Supplement: Appendix — Additional and methods for study of whole-genome sequencing of Salmonella enterica subtypes Mississippi and Typhimurium DT160, Tasmania, Australia, and New Zealand. [file 18-1811-Techapp-s1.pdf]

Article DOI: https://doi.org/10.3201/eid2509.181811

Whole-Genome Sequencing of *Salmonella* Mississippi and Typhimurium Definitive Type 160, Australia and New Zealand

Appendix

Methods

Salmonella Mississippi Sampling Strategy

We sampled from 529 *Salmonella* Mississippi human isolates with a Tasmanian postcode at the Microbiological Diagnostic Unit Public Health Laboratory (MDU PHL) and isolated between 2011 and 2015. We excluded any isolate with an indication of overseas travel (n = 2), sorted by isolation date, and then sampled every 1 in 15 isolates (n = 36). When comparing the characteristics of sampled isolates to the population, there was a higher proportion of males in the sample (Appendix Table 1).

Therefore, we re-sampled, sorting by isolation date and sampling every 2nd in 15 isolates (n = 36). As the characteristics of this sample were closer to the population, they were chosen for sequencing (Appendix Table 2, Appendix Figures 2, 3).

We also sampled from 74 isolates from other states that were isolated between 2011 and 2015 and at MDU PHL. We excluded isolates with an indication of overseas acquisition (n = 12). We selected the 1 isolate available from the Australian Capital Territory (ACT), 1 from South Australia (SA), and 1 from Queensland (Qld), all with travel to Tasmania reported. There were 6 isolates with a New South Wales postcode and 2 isolates with a Western Australia postcode. We randomly selected 1 from each of these states, both with travel to Tasmania reported. There were 51 isolates with a Victorian postcode. We sorted by postcode and selected 1 in 5, sampling 10 isolates from Victoria. The characteristics of cases with selected isolates compared to all isolates in other states is in Appendix Table 3.

In addition, we opportunistically sampled 12 *Salmonella* Mississippi human isolates that were at Queensland Health Forensic and Scientific Services, which were isolated between 2004 and 2009. Of these, 42% were from males, and the median age was 33.5. Some isolates were from cases involved in an unpublished 2008 case–control study. The isolates were sent to MDU PHL for sequencing.

Additional Genomics Information

As there is no publicly available complete *Salmonella* Mississippi genome, a local reference was assembled using one of the MDU PHL *Salmonella* Mississippi isolates. Preliminary analysis showed the *Salmonella* Mississippi genomes had similar QC stats and the oldest sequenced strain (AUSMDU00020775) was selected (2000). Illumina reads of the isolate were assembled using Unicycler (*1*) using a minimum contig length on 200 bp. The resulting assembly was used as the reference for the analysis of the core genome. AUSMDU00020775 assembly comprised 92 contigs, with total number of reference bases 4672632 and an N50 of 366033.

We looked at 11 publicly available draft assemblies of *Salmonella* Mississippi (*2–4*). The sequence type (ST) of the publicly available draft assemblies were determined using *mlst* (v2.11-dev) (https:/github.com/tseemann/mlst) in conjunction with the "senterica" MLST scheme. Of note, the majority of the public and the MDU PHL *Salmonella* Mississippi isolates did not have a known ST. One publicly available isolate (Mississippi_BCW_4007) (*4*) had no alleles the same as the other *Salmonella* Mississippi genomes, indicative that this was a highly divergent strain of *Salmonella* Mississippi. This isolate was excluded from further analysis. Nine of the remaining included publicly available draft assemblies were from soil or Takahe from 2011–2013 in New Zealand (*3*) and 1 was from a human in 2010 from the United States of America (*2*).

Results

Single-Nucleotide Polymorphisms (SNPs) between Australian and New Zealand Isolates

While there were only 9 publicly available *Salmonella* Mississippi isolates from New Zealand, there were a large number of SNPs between these isolates and those from Australia (Appendix Figure 3). There was also considerable heterogeneity within the Australian isolates, with a maximum of 649 SNPs between 2 isolates.

Comparatively, there was much less variability among the Australian and New Zealand *Salmonella* Typhimurium DT160 isolates (Appendix Figure 4).

Violin plots were made using Stata SE 14, with the vioplot package.

Selected Risk Factors for *Salmonella* Mississippi and *Salmonella* Typhimurium DT160

We compared data on risk factors for *Salmonella* Mississippi and *Salmonella* Typhimurium DT160 cases in the week before the onset of illness using a 2-sample test of proportions (Appendix Table 4).

Salmonella Typhimurium DT160 Australian Case Animal Contact

Of the 88% (36/41) of Australian *Salmonella* Typhimurium DT160 cases that reported direct animal contact, animals included dogs (n = 27), cats (n = 18), chickens (n = 12), birds (n = 6), possum (n = 3), wallabies or kangaroos (n = 3), horses (n = 3), sheep (n = 2), rabbits (n = 2), and 1 each of cows, goats, pigs, fish, guinea pigs, deer, bandicoot, and worms and snails.

References

1. Wick RR, Judd LM, Gorrie CL, Holt KE. Unicycler: resolving bacterial genome assemblies from short and long sequencing reads. PLOS Comput Biol. 2017;13:e1005595. PubMed https://doi.org/10.1371/journal.pcbi.1005595

2. Gupta R, Schmidtke A, Sabol A, Castillo D, Ribot E, Trees E. *Salmonella enterica* subsp. *enterica* serovar Mississippi str. 2010K–1406, whole genome shotgun sequencing project. Acession ALPQ000000001. GenBank. 2012 [cited 2018 Apr 23]. https://www.ncbi.nlm.nih.gov/nuccore/ALPQ00000000.1

3. Grange ZL, Biggs PJ, Rose SP, Gartrell BD, Nelson NJ, French NP. Genomic epidemiology and management of *Salmonella* in island ecosystems used for takahe conservation. Microb Ecol. 2017;74:735–44. PubMed https://doi.org/10.1007/s00248-017-0959-1

4. den Bakker HC, Moreno Switt AI, Govoni G, Cummings CA, Ranieri ML, Degoricija L, et al. Genome sequencing reveals diversification of virulence factor content and possible host adaptation in distinct subpopulations of *Salmonella enterica*. BMC Genomics. 2011;12:425. PubMed https://doi.org/10.1186/1471-2164-12-425

Appendix Table 1. Sex and median age of cases with sampled isolates and all *Salmonella* Mississippi isolates, Tasmania, 2011–2015, MDU PHL, sample 1

Variable	All isolates	Sampled isolates
% M	47	64
Median age, y	45	50

Appendix Table 2. Sex and median age of cases with sampled isolates and all *Salmonella* Mississippi isolates, Tasmania, 2011–2015, MDU PHL, sample 2

Variable	All isolates	Selected isolates
% M	47	53
Median age, y	45	42

Appendix Table 3. Sex and median age of cases with sampled isolates and all *Salmonella* Mississippi isolates, mainland Australia 2011–2015, MDU PHL

Variable	All isolates	Selected isolates
% M	40	38
Median age, y	45	54

Appendix Table 4. *Salmonella* Mississippi and *Salmonella* Typhimurium DT160 cases who answered yes to risk factor questions of those who answered the question, Australia

Variable	*Salmonella* Mississippi, no. (%)	*Salmonella* Typhimurium DT160, no. (%)	p value
Bushwalking	2/25 (8)	3/40 (7.5)	0.94
Camping	2/26 (8)	0/40 (0)	0.07
Gardening	4/25 (16)	9/41 (22)	0.56
Swimming	4/25 (16)	7/39 (18)	0.84
Drinking from an untreated raw water source	14/23 (61)	12/30 (40)*	0.1
Animal contact	19/28 (68)	36/41 (88)	0.04

*2/12 (17%) reported boiling their water before drinking.

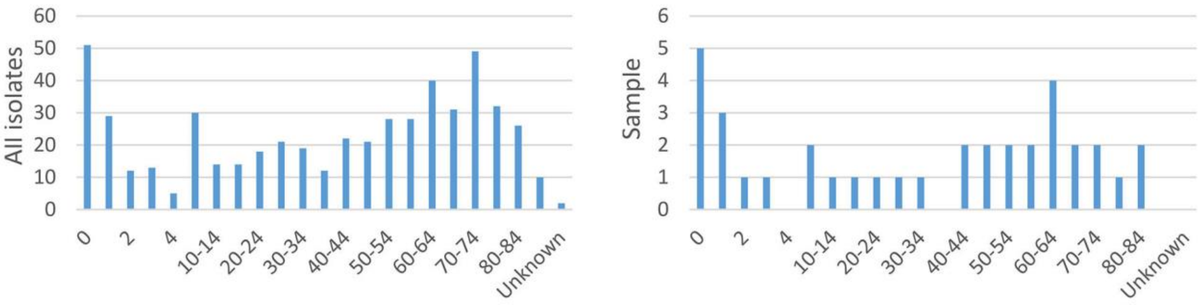

Appendix Figure 1. Age distribution of all *Salmonella* Mississippi cases with isolates, Tasmania, Australia, 2011–2015, Microbiological Diagnostic Unit Public Health Laboratory, sample 2.

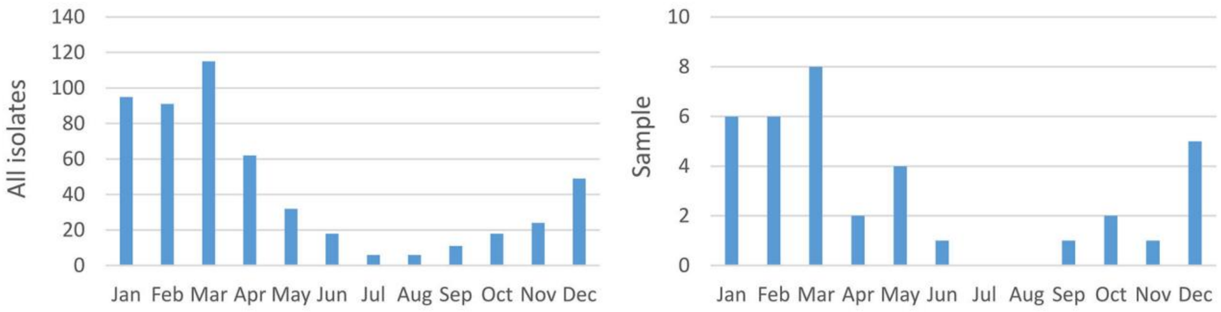

Appendix Figure 2. Month of isolation of all *Salmonella* Mississippi cases with isolates, Tasmania, Australia, 2011–2015, Microbiological Diagnostic Unit Public Health Laboratory, sample 2.

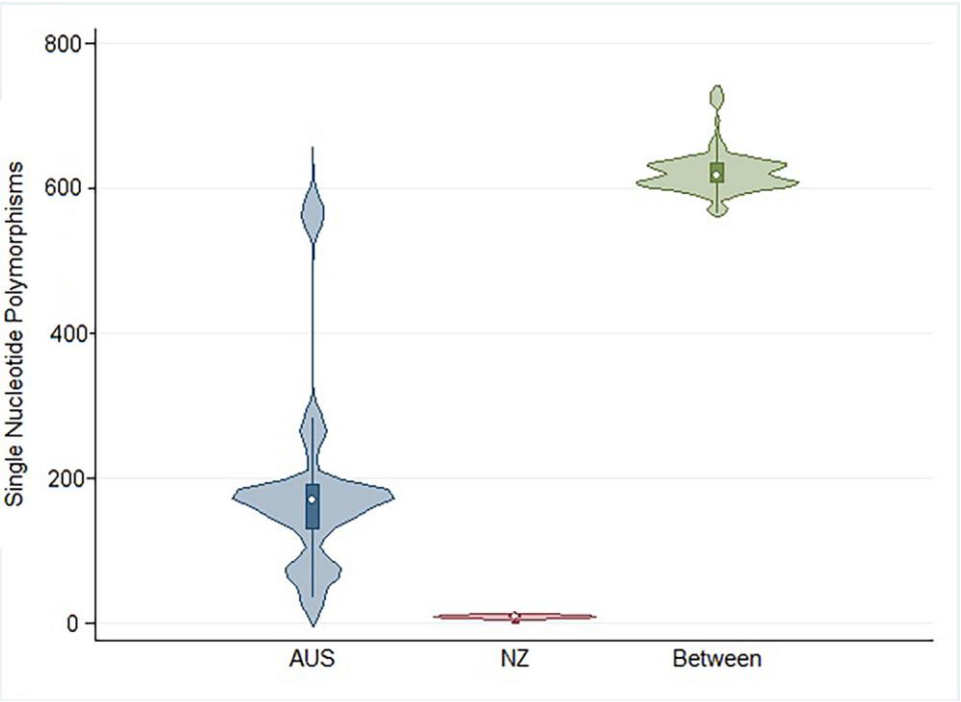

Appendix Figure 3. Violin plot of single nucleotide polymorphisms within Australian and New Zealand clades and between them, *Salmonella* Mississippi, 2008–2015.

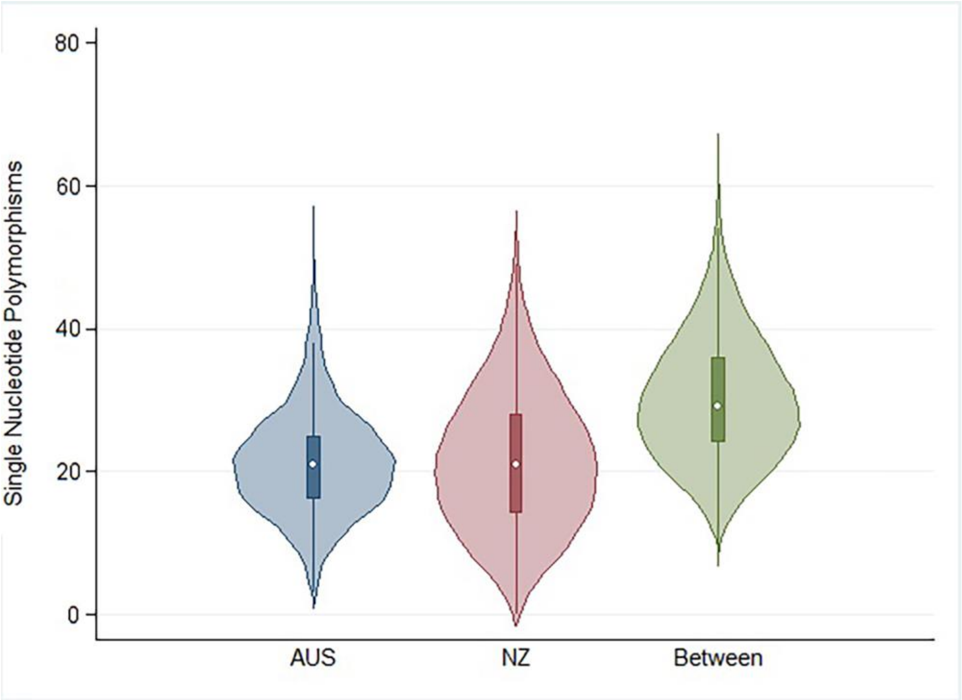

Appendix Figure 4. Violin plot of single nucleotide polymorphisms within Australian and New Zealand clades and between them, *Salmonella* Typhimurium definitive type 160, 1998–2016.
